# Disparities of time trends and birth cohort effects on invasive breast cancer incidence in Shanghai and Hong Kong pre- and post-menopausal women

**DOI:** 10.1186/s12885-017-3359-5

**Published:** 2017-05-23

**Authors:** Feng Wang, Lap Ah Tse, Wing-cheong Chan, Carol Chi-hei Kwok, Siu-lan Leung, Cherry Wu, Oscar Wai-kong Mang, Roger Kai-cheong Ngan, Mengjie Li, Wai-cho Yu, Koon-ho Tsang, Sze-hong Law, Xiaoping Miao, Chunxiao Wu, Ying Zheng, Fan Wu, Xiaohong R. Yang, Ignatius Tak-sun Yu

**Affiliations:** 1 JC School of Public Health and Primary Care, the Chinese University of Hong Kong, Sha Tin, Hong Kong SAR China; 2 Department of Surgery, North District Hospital, Sheung Shui, Hong Kong SAR China; 3 Department of Oncology, Princess Margaret Hospital, Kwai Chung, Hong Kong SAR China; 4 Department of Surgery, Pamela Youde Nethersole Eastern Hospital, Chai Wan, Hong Kong SAR China; 5 Department of Pathology, North District Hospital, Sheung Shui, Hong Kong SAR China; 6 Hong Kong Cancer Registry, Hospital Authority, Yau Ma Tei, Hong Kong SAR China; 7 Department of Medicine and Geriatrics, Princess Margaret Hospital, Kwai Chung, Hong Kong SAR China; 8 Department of Pathology, Yan Chai Hospital, Tsuen Wan, Hong Kong SAR China; 9 Department of Surgery, Yan Chai Hospital, Tsuen Wan, Hong Kong SAR China; 10 Department of Epidemiology and Biostatistics, Tongji School of Public Health, Huazhong University of Science and Technology, Wuhan, China; 11 Shanghai Municipal Center for Disease Control & Prevention, Shanghai, China; 12 Genetic Epidemiology Branch, Division of Cancer Epidemiology & Genetics, National Cancer Institute, National Institutes of Health, Bethesda, MD USA; 13 School of Public Health and Primary Care, the Chinese University of Hong Kong, Prince of Wales Hospital, 4/F, Sha Tin, N.T, Hong Kong SAR China

**Keywords:** Breast cancer, Annual percentage change, Incidence, Age-period-cohort modeling

## Abstract

**Background:**

Breast cancer is the leading cause of cancer morbidity among Shanghai and Hong Kong women, which contributes to 20–25% of new female cancer incidents. This study aimed to describe the temporal trend of breast cancer and interpret the potential effects on the observed secular trends.

**Methods:**

Cancer incident data were obtained from the cancer registries. Age-standardized incidence rate was computed by the direct method using the World population of 2000. Average annual percentage change (AAPC) in incidence rate was estimated by the Joinpoint regression. Age, period and cohort effects were assessed by using a log-linear model with Poisson regression.

**Results:**

During 1976–2009, an increasing trend of breast cancer incidence was observed, with an AAPC of 1.73 [95% confidence interval (CI): 1.54–1.92)] for women in Hong Kong and 2.83 (95% CI, 2.26–3.40) in Shanghai. Greater upward trends were revealed in Shanghai women aged 50 years old or above (AAPC = 3.09; 95% CI, 1.48–4.73). Using age at 50 years old as cut-point, strong birth cohort effects were shown in both pre- and post-menopausal women, though a more remarkable effect was suggested in Shanghai post-menopausal women. No evidence for a period effect was indicated.

**Conclusions:**

Incidence rate of breast cancer has been more speedy in Shanghai post-menopausal women than that of the Hong Kong women over the past 30 years. Decreased birth rate and increasing environmental exposures (e.g., light-at-night) over successive generations may have constituted major impacts on the birth cohort effects, especially for the post-menopausal breast cancer; further analytic studies are warranted.

**Electronic supplementary material:**

The online version of this article (doi:10.1186/s12885-017-3359-5) contains supplementary material, which is available to authorized users.

## Background

Breast cancer is now the most common cancer in women worldwide, especially in the metropolises [1]. Hong Kong and Shanghai, being the most westernized and urbanized cities in China, have presented the highest incidences of breast cancer among China. Breast cancer is the top one malignancy and accounts for 20–25% of new cancer cases among women in these two cities [2, 3]. Although the incidence rate of breast cancer in Hong Kong and Shanghai are nearly 2-folds lower than that in the United States [4], these rates have increased faster than global rate [5].

In the most recent decades, Hong Kong and Shanghai successively underwent an accelerating socioeconomic development, which is reflected in many aspects including an adoption of western lifestyle, changes of reproductive pattern and ageing of their population [6, 7]; these are the established risk factors that may have contributed greatly to the increasing rate of breast cancer incidence. Given different health policy and political background between Shanghai and Hong Kong, in particular the performance of unique birth control policy in mainland China over the past 30 years, the risk patterns of breast cancer for women in these two cities may exist large discrepancies.

Previous time trend studies on breast cancer were based on the overall analysis that might have masked the actual trends of pre-menopausal (early age onset) and post-menopausal breast cancer, between they represent different disease entities with various etiology [4]. A separate analysis of time trend by menopausal status is thus regarded as proper for disclosure of actual risk patterns. This study described the temporal trend of breast cancer among Hong Kong and Shanghai women and assessed the potential effects contributing to the increasing trend in pre- and post-menopausal breast cancers by using a novel approach of age-period-cohort modeling (APC) developed by Rutherford et al. [8].

## Methods

### Data sources

Data on newly diagnosed invasive breast cancer were retrieved from the Hong Kong Cancer Registry (HKCaR) (Data is available: http://www3.ha.org.hk/cancereg/) and Shanghai Cancer Registry (SHCaR) (Data is available from the ‘Tumor Annual Report of Shanghai’), which both are accredited members of the International Association of Cancer Registries (IACR). Briefly, these two cancer registries are population-based cancer registries. The completeness and quality of data was reported an over 95% coverage of most cancers for HKCaR, and coverage of cancer cases registered by SHCaR is nearly 100% [2, 3].

Population data during the corresponding period was obtained from the Hong Kong Census and Statistics Department (Data is available: http://www.censtatd.gov.hk/hkstat/sub/so20.jsp) and Shanghai Statistics Bureau (Data is available from the ‘Shanghai Statistical Yearbook’). Mid-year population data were employed in the calculation of the incidence rate.

### Statistical analysis

Age-standardized incidence rates were calculated by using the direct method and taking the WHO world standard population 2000 as the reference population. Because of the small number of breast cancer diagnosed in women younger than 20 years, these cases were excluded from all analyses. We stratified the cases into pre- and post-menopause subgroups using a cut-point of 50 years old that is the median age at menopause among Chinese women [9]. So all grouped age-standardized rates were calculated as truncated rates. Trend of breast cancer incidence was evaluated by the Joinpoint-Regression Program (Version 4.1.0, Statistical Research and Applications Branch, National Cancer Institute, USA). Joinpoint regression identifies statistically significant trend change points (joinpoints) and the rate of change (average annual percent change, AAPC).

Age-period-cohort modeling is a useful framework to understand the temporal trend of key diseases’ prevalence and estimate the effects of three time-dependently scales - age, diagnostic period and birth cohort. A fundamental issue of APC is the linear dependence among age, period and cohort effects, which limited to obtain the unique effect of each time-dependent variable [10, 11]. We employed a new age-period-cohort modeling method which developed by Rutherford et al. [8] to investigate the effects of age, period and cohort on the incidence of breast cancer. This novel method overcomes the over-dispersion amongst time-dependent variables by fitting a log-linear model with a Poisson distribution to obtain ‘unbiased’ age, period and cohort effects in the same model. R-statistical software was used for trend analysis (Epi package version1.1.67, R version 3.1.1), while other statistical analyses were performed with the Statistical Package for the Social Sciences (SPSS) version 20.0 (IBM Corp, Somers, NY). A *p* value of <0.05 was regarded as statistically significant.

## Results

**Fig. 1:**
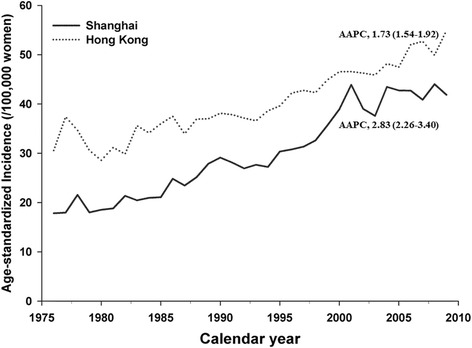
Age-standardized incidence rates for breast cancer among Hong Kong and Shanghai women, 1976–2009

The present analyses were based on 48,367 (Hong Kong) and 44,344 (Shanghai) cases of invasive breast cancer reported from all hospitals from 1976 to 2009. The age standardized incidence rates of breast cancer in Hong Kong women increased from 28.6 to 54.9/100,000 women which were always higher than those in Shanghai women (from 17.8 to 44.0/100,000 women) during the study period (Fig. 1). The AAPC were 1.73 [95% confidence interval (95% CI), 1.54–1.92] for Hong Kong which is lower than that for Shanghai (AAPC = 2.83, 95% CI, 2.26–3.40) (*p* < 0.01). Incidence rates by age and diagnostic period are shown in Table 1.

**Table 1:** Age-specific incidence rates (per 100,000 women) of breast cancer in Hong Kong and Shanghai, 1976–2009

Age group	Shanghai							Hong Kong						
	1976–80	1981–85	1986–90	1991–95	1996–00	2001–05	2006–09	1976–80	1981–85	1986–90	1991–95	1996–00	2001–05	2006–09
20–24	0.76	0.64	0.89	0.91	0.52	1.00	1.13	1.55	1.38	1.73	1.97	1.25	0.84	1.14
25–29	3.11	2.80	3.90	3.38	4.52	5.78	3.88	5.84	6.68	6.24	5.24	7.13	5.85	5.84
30–34	11.47	11.15	13.78	15.03	11.95	12.20	12.80	15.11	15.90	17.96	19.30	21.30	18.19	21.45
35–39	21.20	26.37	30.68	33.48	33.54	27.19	29.98	34.64	35.59	43.28	44.22	46.90	46.39	49.89
40–44	36.52	47.01	61.33	53.67	72.01	75.18	65.63	43.07	52.37	78.59	71.78	88.30	94.46	98.95
45–49	43.78	45.96	70.20	78.51	87.79	121.45	117.36	65.34	73.62	78.02	98.24	113.95	129.20	144.66
50–54	45.92	48.34	64.86	71.89	97.14	124.51	125.84	73.28	71.33	78.40	88.81	115.59	124.18	152.04
55–59	48.40	45.86	62.41	71.32	90.87	120.44	123.09	79.21	90.37	77.06	84.47	116.14	136.75	148.46
60–64	58.09	56.62	61.63	75.79	96.28	102.66	134.51	103.12	92.23	95.78	90.88	98.08	120.66	150.10
65–69	47.23	59.82	69.06	70.55	87.00	106.73	127.36	133.94	109.43	121.41	101.61	103.75	113.68	129.84
70–74	53.67	58.14	72.41	79.57	89.75	116.53	126.62	113.92	131.46	123.06	118.38	118.12	118.96	126.27
75–79	44.84	60.48	64.76	70.76	88.31	109.77	120.91	120.63	120.82	131.89	130.87	132.63	130.41	130.53
80–84	53.64	62.68	67.05	71.68	91.93	116.32	101.84	87.06	114.12	142.96	162.43	167.18	150.61	148.07
≥85	62.86	44.27	53.00	60.90	80.62	83.14	96.30	122.91	114.09	136.32	156.57	172.06	149.22	146.81
Overall	18.78	20.53	26.08	28.06	33.86	41.34	42.35	32.35	33.34	36.69	37.95	43.73	46.87	52.38

**Fig. 2:**
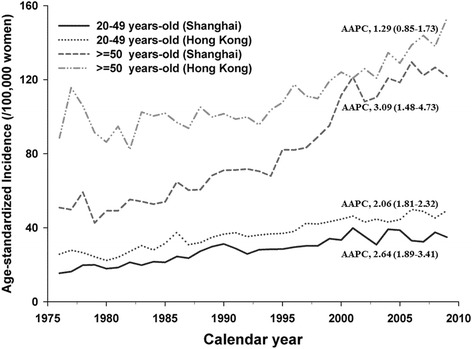
Age-specific incidence rates for breast cancer by 50 years-old among Hong Kong and Shanghai women, 1976–2009

The incidence trends were inconsistent in Hong Kong and Shanghai women after stratified by age (Fig. 2). The increasing trends were similar for Hong Kong and Shanghai women aged among 20–49 years-old (*p* = 0.58), with an AAPC of 2.06 (95% CI, 1.81–2.32, Hong Kong) and 2.64 (95% CI, 1.89–3.41, Shanghai), respectively. However, the increasing trend of Shanghai women who were 50 years-old or above was more speedy (AAPC = 3.09, 95% CI, 1.48–4.73) than that of Hong Kong women (AAPC = 1.29, 95% CI, 0.85–1.73) (*p* < 0.01).

**Fig. 3:**
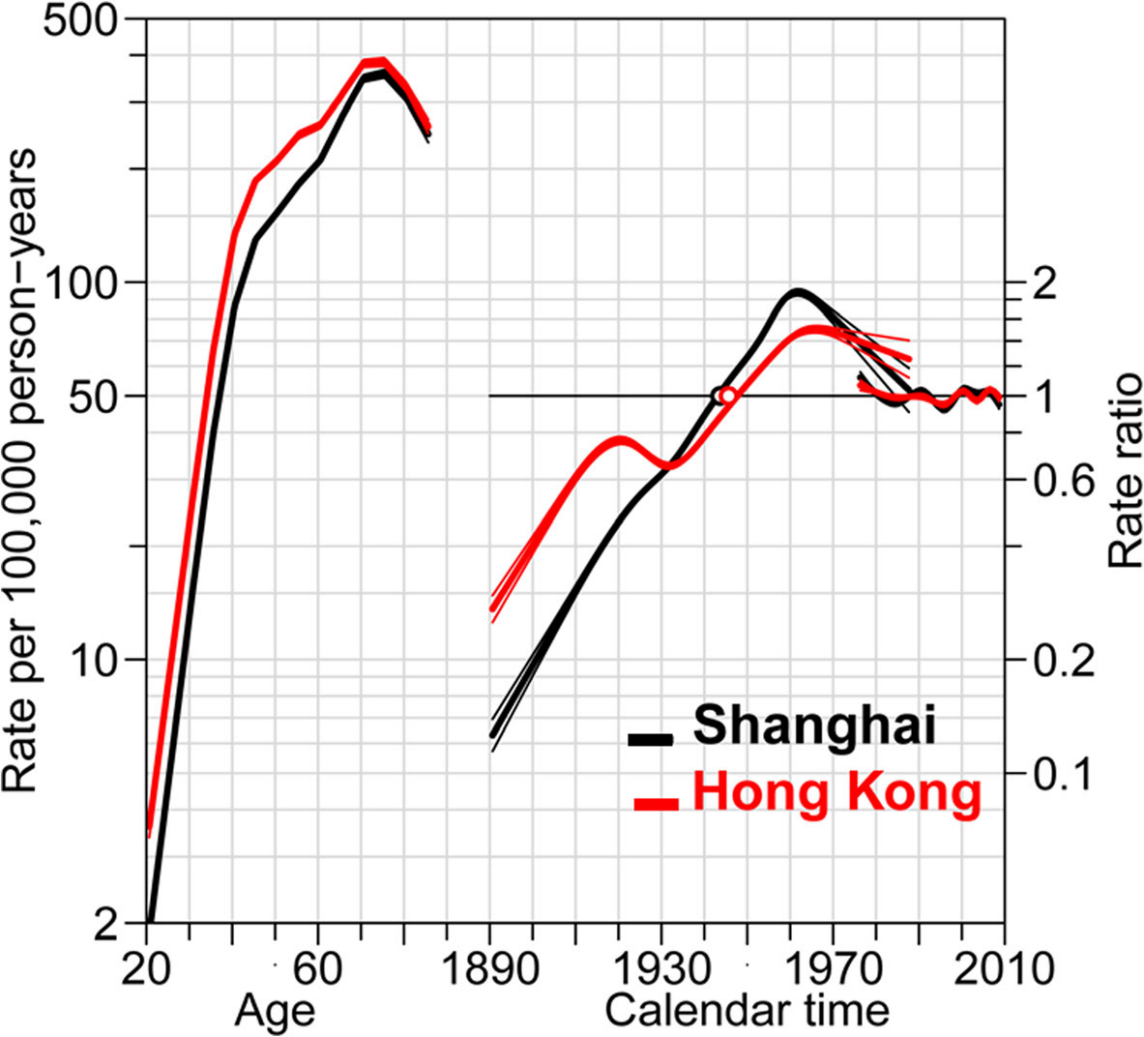
Age-period-cohort effect analysis of breast cancer among Hong Kong and Shanghai women. Curves (*red and black*) in left side showed age-specific incidences of breast cancer; Curves in middle part showed cohort effects; curves in right side showed period effects

**Fig. 4:**
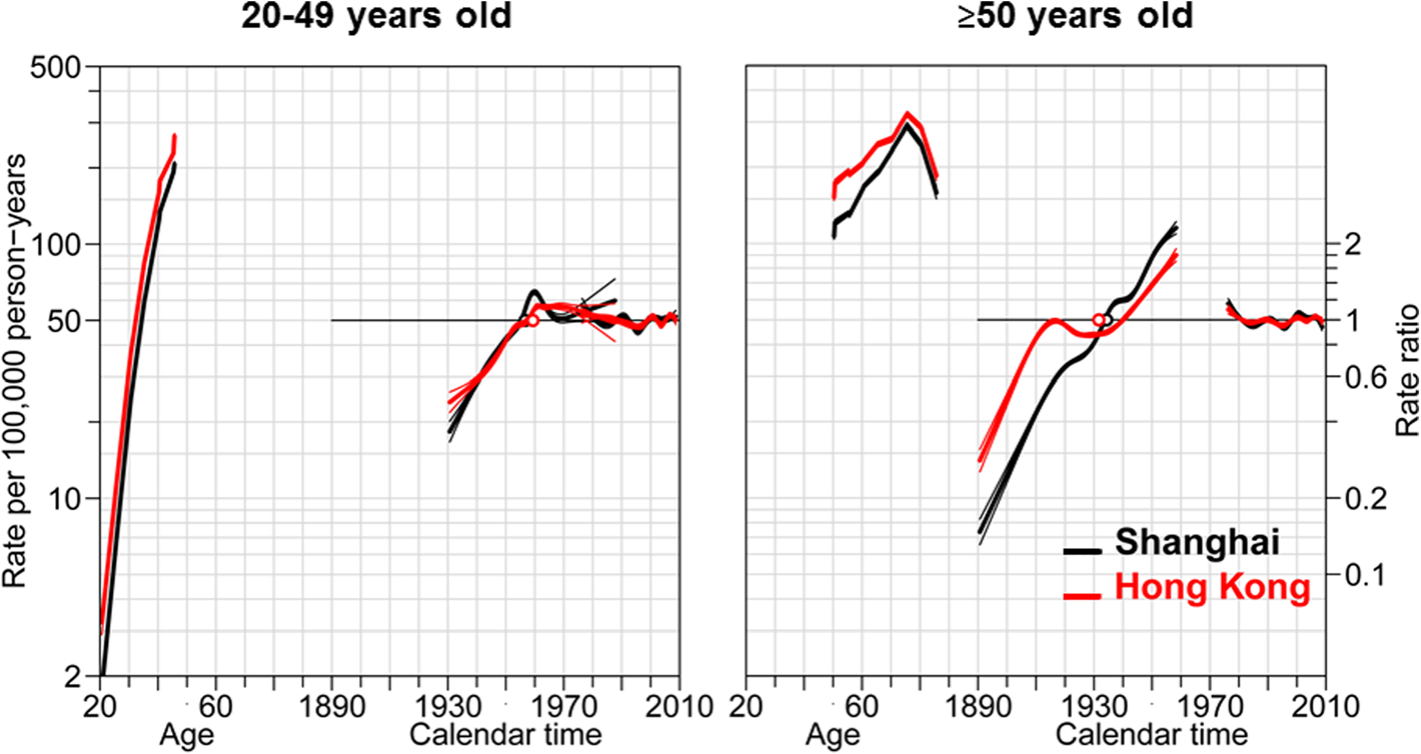
Age-period-cohort trends analysis by 50 years-old among Hong Kong and Shanghai women. Curves (*red and black*) in left side showed age-specific incidences of breast cancer; Curves in middle part showed cohort effects; curves in right side showed period effects

Results of the goodness-of-fit for the models of age, period and cohort are shown in Fig. 3 and Additional file 1: Table S1. Age and cohort effects contributed significantly to the increasing trends, although the contribution of period effect couldn’t be entirely neglected. The age and period effects on breast cancer trends were similar between Hong Kong and Shanghai women in both groups. For the younger cases (age 20–49 years old), the cohort effect of Hong Kong women was quite similar as that of Shanghai women. Their relative risks (RR) increased in a similar magnitude for women born before 1961 (left part of Fig. 4, using the 1961 birth cohort as reference). For the elder cases (age ≥ 50 years old), the cohort effect of Shanghai women was more remarkable that of Hong Kong women, with a higher RR (0.15 to 2.31) for the most recent generation in Shanghai women than their counterparts in Hong Kong (0.28 to 1.80) (right part of Fig. 4, using the 1926 birth cohort as reference). There was no evidence for a significant change of relative risk for the period effects.

## Discussion

Breast cancer is the most common malignant tumor among Chinese urban women. The highest rates occur in eastern coastal urban areas that are socioeconomically well developed [1]. Our data demonstrated an average annual increase of 1.73% and 2.83% in the incidence rates of invasive breast cancer among Hong Kong and Shanghai women for the calendar period 1976–2009. Of note, this increase was more remarkable in post-menopausal women of Shanghai (AAPC = 3.09), whilst the trends of pre-menopausal breast cancer incidence rate were similar among Hong Kong and Shanghai women. Stronger birth cohort effects were indicated in the most recent generations for the post-menopausal breast cancer cases in Shanghai than those of the Hong Kong.

The increasing trends of breast cancer incidences among Hong Kong and Shanghai women are parallel with the fast socioeconomic development and urbanization of these cities. The strong birth cohort effect observed in Shanghai and Hong Kong women was consistent with studies conducting in other Asia countries [12–15]. Decreased birth rate and late age at first birth, westernized lifestyle and possible environmental exposures [e.g., endocrine disrupting chemicals (EDCs)] between women born in successive cohorts may have contributed to the upward trends in incidence rate of breast cancer [16]. With the development of economics, the reproductive pattern of Hong Kong and Shanghai women changed significantly in the recent half century. In Hong Kong, age at first birth postponed for about 5 years (25.1 years old in 1981 *v.s.* 30.0 years old in 2011); Parity decreased from 3.73/woman born in 1936 to 1.56/woman born in 1961, which linked to an increase in the nulliparity rate from 8.1% for women born in 1936 to 22.5% for women born in 1961 [17]. As an indicator of productive pattern, the total fertility rate (TFR) of Hong Kong decreased to 1.06 in 2009. Similar situation also occurred among Shanghai women [17, 18]. The urban Shanghai has the lowest TFR worldwide [1]. It was reported that decreased numbers of births per woman have been associated with an increased risk of breast cancer (odd ratio 1.45) for post-menopausal women in Shanghai [19]. The low birth rate observed in both Shanghai and Hong Kong women over the past 30 years might have contributed greatly to the cohort-driven trend of post-menopausal breast cancer in later years. Considering there were two decades later between Hong Kong and Shanghai whose TFR was less than 1, it could be projected that the incidence trend might keep increasing in the post-menopausal women of Hong Kong.

Obesity, lack of physical activity and excessive alcohol also are known risk factors of breast cancer [16]. The prevalence of overweight slightly increased from 33.7% in 1995 to 35.9% in 2005 among Hong Kong women [20], while more than one third of female adults were overweight or obesity in Shanghai [21]. On the other hand, the alcohol drinking rate of women increased 5.1% from 2005 to 2010 (19.5% and 24.6%, respectively) in Hong Kong [20]; meanwhile, a survey in 2007 showed that less than 50% female attended moderate physical activity once per week. These changes in lifestyles may also yield impacts on the increasing trends of breast cancer incidence.

Emerging environmental risk factors that catch the public’s attention are the disrupted circadian rhythm induced by light at night (LAN) and exposure of environmental endocrine disruptors. Night shift work, as a typical surrogate of circadian rhythm disruption, has been classified as the Group 2A carcinogen by the International Agency for Research on Cancer (IARC) [22]. Prolonged exposure to night shift work is evident to be associated with an increased risk of female breast cancer, showing a dose-response relationship with night shifts experienced [23]. The hypothesized mechanism is that LAN suppresses the production of melatonin which increased incident breast cancer [24]. Apart from high prevalent night shift work, Hong Kong and Shanghai have high nighttime illumination. And it has very high building density and close proximity of commercial and residential premises. The outdoor light can easily penetrate into bedrooms when people turn off their household electric light. LAN exposure might be a potential risk of female breast cancer.

Evidence showed that prenatal exposure to environmental endocrine disruptors such as dioxin and polychlorinated biphenyls (PCBs) increased risk of human breast tumors [25]. Meanwhile, serum level of dioxin was positively related with breast cancer incidence [26]. Environmental dioxin and PCBs can accumulate in animal fatty tissues and readily be absorbed into human body, which is the major source of human exposure to these compounds. Fish consumption is very high in the eastern coastal areas of China. A recent report documented that about 3.1% Hong Kong population consumed exceeded dioxin and dioxin-liked PCBs from food, in particular fish [27], which might be partly contributed to the increasing trend of breast cancer in Hong Kong female population.

Mammography screening as a diagnostic practice may contribute to the period effect, but it should not be a major concern. Hong Kong started to promote mammography screening from earlier 1990s, but there was no population screening till now [28]. The less intensive promotion of mammography among young women might artificially increase the breast cancer incidence at the young age group but no obvious upward in period effect was suggested. Moreover, the period curvature was plateau, which further supports that the increasing trend is more likely to reflect the actual secular trend rather than an artificial one. A significant change was seen in the ductal carcinoma in situ (DCIS). The diagnosed DCIS increased from <1% in 1980s to 11% in 2010s among all breast cancer patients (unpublished data). Nevertheless, DCIS wasn’t included in the current analyses.

A deceleration for birth cohort later than 1960 in Hong Kong was reported by Wong IO et al., who explained this by the cap in the population effects from socioeconomic development [29]. We observed a similar trend to that of the Shanghai women, and thus considered that a decreased cohort effect for the birth cohort 1960 and thereafter might be an artificial issue that may not reflect the socioeconomic development, because women of these birth cohorts were too young to reach the peak age of breast cancer occurrence.

## Conclusions

This study revealed that the time trends of breast cancer incidence kept increasing among Hong Kong and Shanghai women. Decreasing birth rates and later age at first birth might have contributed to the increasing rate, in particular the post-menopausal breast cancer incidence. Moreover, emerging environmental risk factors, such as disruption of circadian rhythm induced by prolonged exposure to light at night and exposure to environmental endocrine disruptors might also contribute to the increasing trends of breast cancer incidence for both Shanghai and Hong Kong women. Nevertheless, hypotheses raised from this descriptive epidemiological research should be confirmed by future analytic studies.

## Additional file

Additional file 1: Table S1. summary statistics of age-period-cohort effect modelling in breast cancer incidences among Hong Kong and Shanghai women, 1976–2009. (DOC 32 kb)

## Abbreviations

APC: Age-period-cohort modeling
HKCaR: Hong Kong Cancer Registry
SHCaR: Shanghai Cancer Registry
IACR: The International Association of Cancer Registries
AAPC: Average annual percent change
RR: Relative risks
EDCs: Endocrine disrupting chemicals
TFR: Total fertility rate
LAN: Light at night
IARC: The International Agency for Research on Cancer
PCBs: Polychlorinated biphenyls
DCIS: Ductal carcinoma in situ
