# Supplementary material for: Disparities of time trends and birth cohort effects on invasive breast cancer incidence in Shanghai and Hong Kong pre- and post-menopausal women
Source: BMC Cancer. 2017 May 23;17:362. doi: 10.1186/s12885-017-3359-5 (PMC5442698; doi:10.1186/s12885-017-3359-5)
Supplement: Additional file 1: Table S1. — summary statistics of age-period-cohort effect modelling in breast cancer incidences among Hong Kong and Shanghai women, 1976–2009. (DOC 32 kb) [file 12885_2017_3359_MOESM1_ESM.doc]

Table S1 summary statistics of age-period-cohort effect modelling in breast cancer incidences among Hong Kong and Shanghai women, 1976-2009

|  | **Resid. Df** | **Df** | **Shanghai** | | |  | **Hong Kong** | | |
| --- | --- | --- | --- | --- | --- | --- | --- | --- | --- |
|  |  |  | **Resid. Dev** | **Deviance** | **Pr (>Chi)** |  | **Resid. Dev** | **Deviance** | **Pr (>Chi)** |
| **Age** | 883 |  | 11362.4 |  |  |  | 6479.9 |  |  |
| **Age-drift** | 882 | 1 | 3967.5 | 7394.9 | < 2.2×10^-16^ |  | 4192.7 | 2287.1 | < 2.2×10^-16^ |
| **Age-Cohort** | 876 | 6 | 3366.7 | 600.8 | < 2.2×10^-16^ |  | 3040.5 | 1152.2 | < 2.2×10^-16^ |
| **Age-Period-Cohort** | 870 | 6 | 3239.2 | 127.5 | < 2.2×10^-16^ |  | 2982.3 | 58.2 | 1.0×10^-10^ |

Resid. Df, residual degree of freedom; Resid. Dev, residual deviance; Pr (>Chi), probability of the Chi-squared distribution.
